# Streptococcal Toxic Shock Syndrome Secondary to Retroperitoneal Necrotizing Fasciitis: A Case Report

**DOI:** 10.7759/cureus.92054

**Published:** 2025-09-11

**Authors:** Masato Takanashi, Takara Inui, Hiroaki Ishida, Hideyuki Terao, Takayuki Murakami

**Affiliations:** 1 Department of Urology, Yokohama City Minato Red Cross Hospital, Yokohama, JPN

**Keywords:** antibacterial agents, necrotizing fasciitis, retroperitoneal space, sepsis, streptococcal infections, streptococcus pyogenes, surgical procedures, toxic shock syndrome

## Abstract

Streptococcal toxic shock syndrome (STSS) is a fulminant septic shock condition that develops abruptly and progresses rapidly due to infection with *Streptococcus pyogenes*. The global incidence of STSS has been increasing, and a wide spectrum of clinical manifestations has been reported. We report a rare case of STSS in a previously healthy 43-year-old woman, secondary to retroperitoneal necrotizing fasciitis. The patient presented with fever and right buttock pain and required intensive care within three days of symptom onset due to rapid disease progression. CT revealed fluid collection and increased fat density in the right retroperitoneal cavity. Emergency lavage and drainage of the right retroperitoneum and paravesical space were performed. Cultures of the hematogenous exudate grew *S. pyogenes*. Following targeted antibiotic therapy, the patient’s condition improved. In STSS, patients often deteriorate suddenly despite having no serious underlying comorbidities, frequently progressing to shock and death. Early consideration of STSS in the differential diagnosis, based on clinical presentation, is critical. If STSS is suspected, prompt surgical intervention, ideally within hours, is essential.

## Introduction

Streptococcal toxic shock syndrome (STSS) has been increasingly reported worldwide in recent years, with its dramatic progression receiving considerable attention in the media. This infectious disease was first systematically described in the 1980s, and sporadic outbreaks have since been reported globally [1]. In addition to fever, STSS is associated with skin and soft tissue inflammation, which can progress to septic shock and multi-organ failure. Even today, the condition remains highly severe, with a reported mortality rate of approximately 30-50% [2]. Although once considered relatively rare, the incidence of STSS has risen markedly, with cases documented in various anatomical sites [3]. We present a case of STSS arising from retroperitoneal necrotizing fasciitis, an extremely uncommon presentation.

## Case presentation

A 43-year-old woman presented to our emergency department on the day of symptom onset (Day 1) with fever and right buttock pain. She had no significant past medical history, was taking no regular medications, and had no known allergies. Her height was 156 cm, weight 46 kg, and BMI 18.9 kg/m².

On examination, she was alert and oriented. Vital signs were temperature 40.0°C, blood pressure 105/56 mmHg, pulse 125 beats/min, respiratory rate 25/min, and oxygen saturation 98% on room air. Physical examination revealed spontaneous pain and tenderness extending from the right groin to the hallux valgus. No erythema, swelling, or visible wounds were noted.

**Figure 1:**
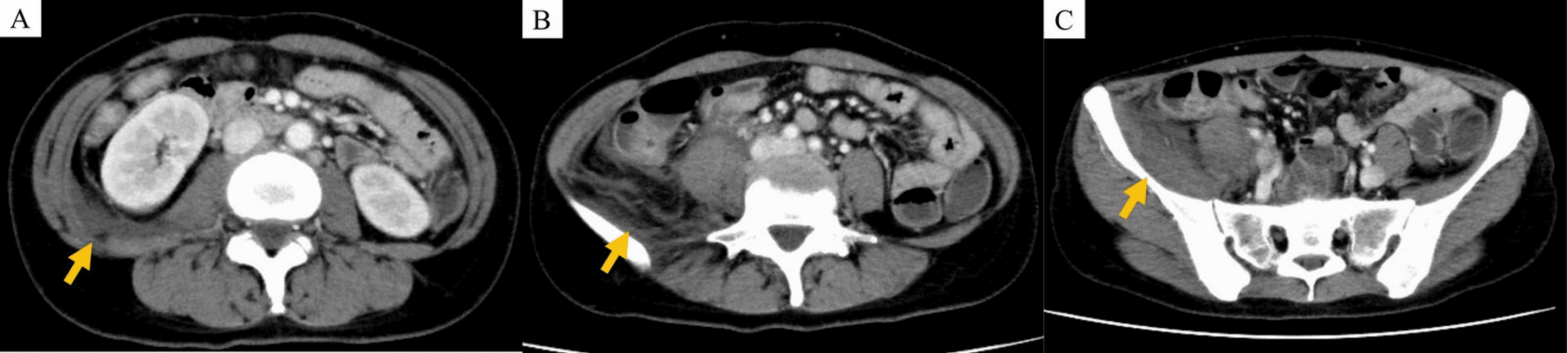
Contrast-enhanced CT findings at the initial visit (A-C) Fluid accumulation and increased fat density in the right retroperitoneal cavity.

Initial laboratory tests showed markedly elevated CRP and leukocytosis, along with renal dysfunction, electrolyte imbalances, coagulation abnormalities, and liver dysfunction. Laboratory findings at the initial visit are shown in Table 1. Blood and urine cultures were negative. Urinalysis was unremarkable. Contrast-enhanced CT demonstrated fluid accumulation and increased fat density in the right retroperitoneal cavity (Figure 1).

**Table 1:** Laboratory findings ALP, alkaline phosphatase; ALT, alanine aminotransferase; APTT, activated partial thromboplastin time; AST, aspartate aminotransferase; Alb, albumin; BUN, blood urea nitrogen; CK, creatine kinase; Cre, creatinine; eGFR, estimated glomerular filtration rate; Glu, glucose; Hb, hemoglobin; LDH, lactate dehydrogenase; Plt, platelet; PT, prothrombin time; T-Bil, total bilirubin; γ-GTP, gamma-glutamyl transferase

Laboratory data	Day 1	Day 3	Reference range and unit
WBC	13.1	15.1	3.5-9.0×10³/μL
Hb	14.8	10.6	13.5-17.5 g/dL
Plt	152	4.5	130-350 ×10³/μL
Na	126	132	138-145 mEq/L
K	3.8	3.5	3.6-4.8 mEq/L
Cl	94	101	101-108 mEq/L
Ca	7.9	6.2	8.8-10.1 mEq/L
BUN	33.4	22	8-20 mg/dL
Cre	2.12	0.96	0.65-1.07 mg/dL
eGFR	21.3	50.6	≥60 ml/min/1.73 m²
LDH	347	394	124-222 U/L
AST	221	85	13-30 U/L
ALT	118	51	10-42 U/L
ALP	130	65	38-113 U/L
γ-GTP	132	91	10-47 U/L
T-Bil	0.6	0.8	0.2-1.2 mg/dL
Alb	3.1	2.2	4.1-5.1 g/dL
PT	15.5	14.1	10-13 seconds
APTT	40.3	58.9	24-36 seconds
CK	499	1049	62-287 U/L
CRP	29.2	33.9	0.00-0.14 mg/dL
Glu	163	68	70-109 mg/dL

**Figure 2:**
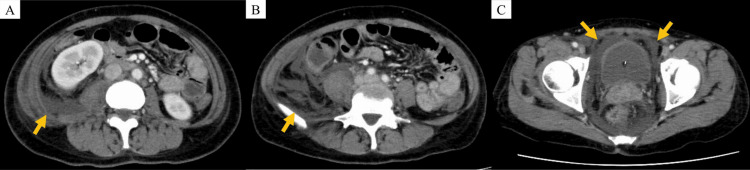
Contrast-enhanced CT findings on Day 3 of hospitalization (A, B) Increased fluid accumulation in the right retroperitoneal space. (C) Extension of fluid collection into the paravesical space.

The patient was admitted with a presumptive diagnosis of retroperitoneal soft tissue infection, and empiric intravenous tazobactam/piperacillin (4.5 g every six hours) was initiated. Despite treatment, the fever persisted. In the early hours of Day 3, she developed hypotension requiring vasopressors. Repeat contrast-enhanced CT revealed increased retroperitoneal fluid extending into the paravesical space (Figure 2), with worsening inflammatory markers (Table 1). Her respiratory status declined later that day, necessitating intubation and intensive care unit admission.

**Figure 3:**
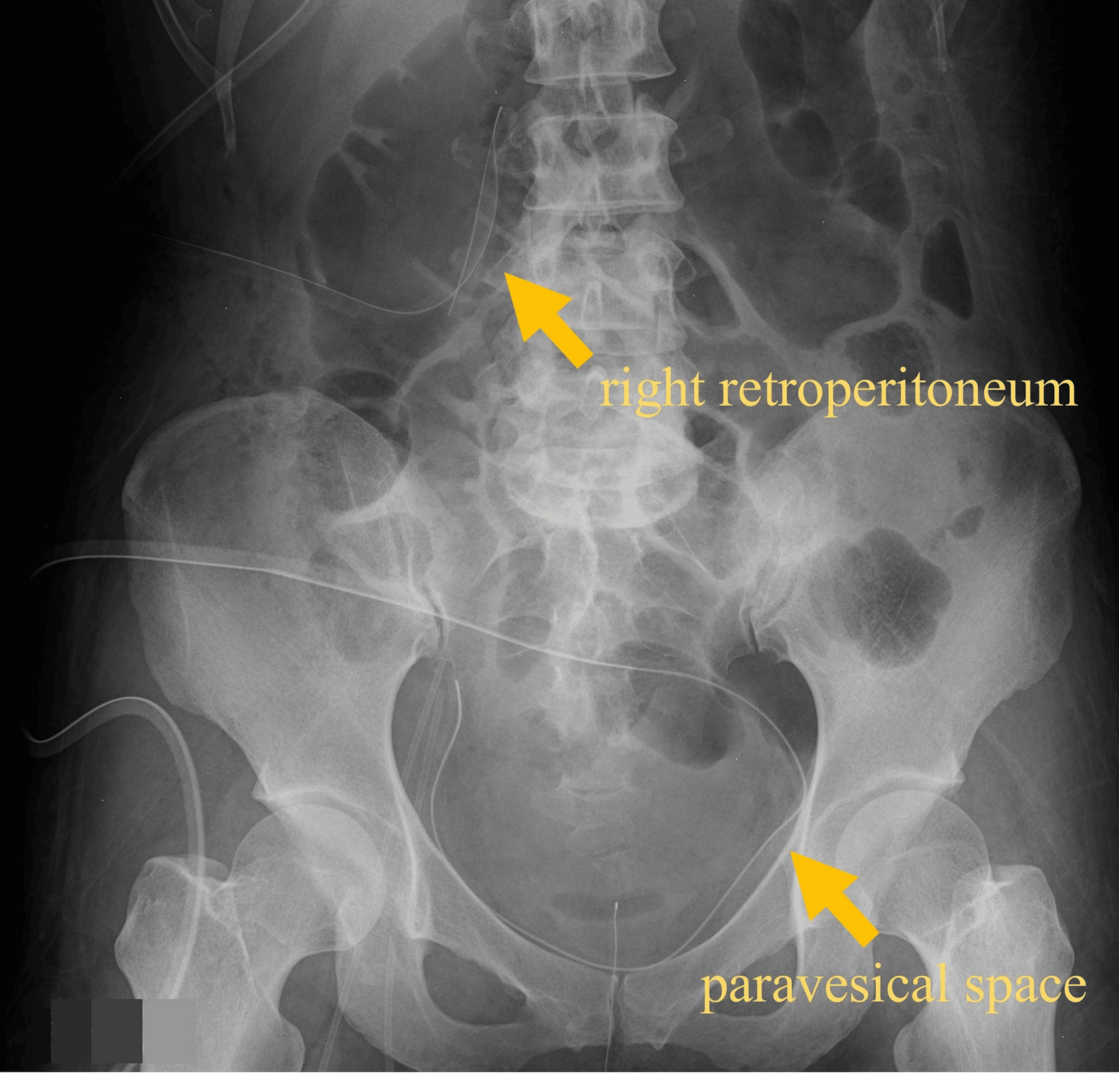
Placement of drainage tubes in the right retroperitoneum and paravesical spaces

That evening, emergency open surgical lavage and drainage of the right retroperitoneum and paravesical space were performed. Retroperitoneal access was achieved via a lateral incision approximately 5 cm above the midaxillary line, followed by a lower midline abdominal incision to access the paravesical space. The retroperitoneal tissue was edematous and inflamed, with pale hematogenous exudate. Intraoperatively, evidence of necrosis was observed in the fascia of the iliopsoas muscle, accompanied by necrosis of the surrounding soft tissues. These necrotic tissues were removed as thoroughly as possible. Drainage catheters were placed in both spaces (Figure 3). The procedure lasted approximately 3 hours, with blood loss of 50 mL. The culture of the exudate grew group A *Streptococcus* (*Streptococcus pyogenes*), which showed susceptibility to all tested antibiotics, including penicillin, clindamycin, and levofloxacin (Table 2). Bacterial identification was performed using matrix-assisted laser desorption/ionization time-of-flight mass spectrometry, MALDI Biotyper (Bruker Daltonik GmbH, Bremen, Germany). Antimicrobial susceptibility testing was conducted with the automated broth microdilution method, VITEK 2 system (bioMérieux, Marcy-l'Étoile, France). Emm typing of the GAS strain was not performed in this case. The patient met the diagnostic criteria for STSS, presenting with hypotension and multi-organ dysfunction, including renal, hepatic, and respiratory impairment, and was therefore diagnosed with the condition secondary to retroperitoneal necrotizing fasciitis.

**Table 2:** Antimicrobial susceptibility test for Streptococcus pyogenes MIC, minimum inhibitory concentration

Antimicrobial agent	MIC (μg/dL)	Interpretation
Ampicillin	≦0.06	Susceptible
Benzylpenicillin	≦0.03	Susceptible
Cefotiam	≦0.5	Susceptible
Cefotaxime	≦0.12	Susceptible
Ceftriaxone	≦0.12	Susceptible
Cefepime	≦ 0.5	Susceptible
Cefozopran	≦0.12	Susceptible
Cefditoren pivoxil	≦0.06	Susceptible
Meropenem	≦0.06	Susceptible
Clavulanate/amoxicillin	≦0.03	Susceptible
Erythromycin	≦0.5	Susceptible
Azithromycin	≦0.12	Susceptible
Clindamycin	≦0.12	Susceptible
Minocycline	≦0.5	Susceptible
Levofloxacin	≦0.12	Susceptible
Vancomycin	≦0.06	Susceptible

**Figure 4:**
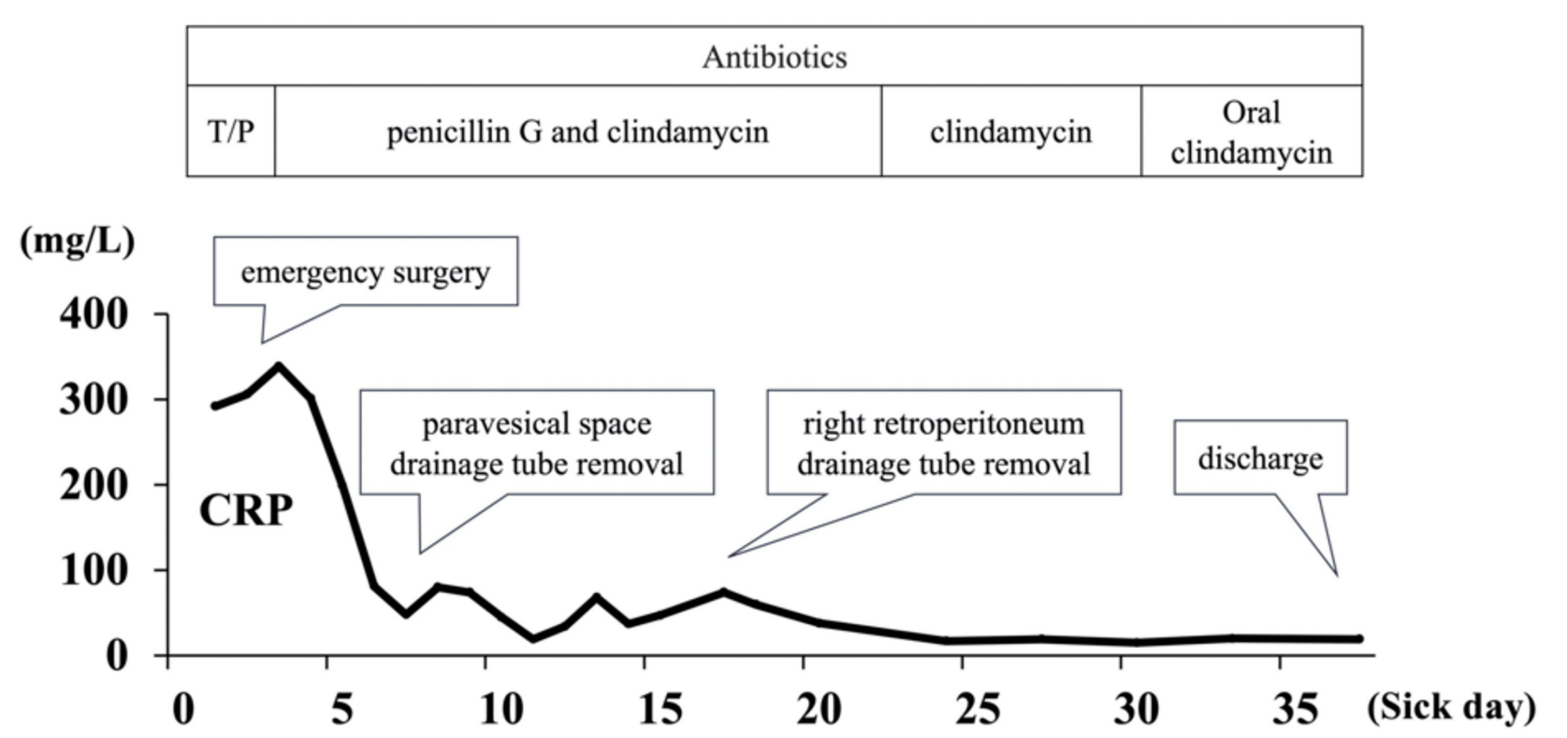
Summary of the overall inpatient clinical course T/P, tazobactam/piperacillin

Postoperatively, antibiotics were de-escalated to intravenous penicillin G (8 million units every eight hours) plus clindamycin (0.6 g every eight hours). The patient’s condition improved rapidly; she was extubated on Day 6 and transferred out of intensive care. Inflammatory markers normalized, and drainage catheters were removed on Days 8 and 17, respectively. She transitioned to oral clindamycin and was discharged on Day 37 without complications. The overall inpatient clinical course is summarized in Figure 4. At follow-up, she remained well with no recurrence for one year.

## Discussion

STSS is a rapidly progressive septic shock syndrome caused by Streptococci [1]. *S. pyogenes* is the main causative agent of STSS. It is a common bacterium that can cause pharyngitis and other infections [4]. Although often contracted via skin breaks or mucosal surfaces, approximately half of STSS cases have no identifiable portal of entry, as in our patient [5]. The precise cause of fulminant streptococcal infection remains unknown. The pathogenesis of STSS is characterized by infectious foci with minimal or no neutrophil infiltration. Previous studies have suggested that the ability of Streptococcus species to evade host immune defenses, particularly neutrophil-mediated responses, may be a critical determinant of both the onset and severity of STSS [6]. Given that outbreaks of STSS are exceedingly rare, it is considered likely that host-related factors, in addition to bacterial virulence determinants, play an important role in disease development.

In the present case, STSS manifested as retroperitoneal necrotizing fasciitis. Although retroperitoneal infections leading to STSS have been rarely reported, we identified two published cases in the literature [7,8]. In both instances, septic shock developed rapidly, necessitating lavage and drainage. Generally, the most frequent initial symptom of STSS is severe pain, which is often followed by sudden and marked swelling and soft tissue necrosis [1]. Patients typically experience an abrupt onset of illness despite having few serious underlying conditions, such as immunodeficiency. Within a short time frame, often within several dozen hours, they may develop acute kidney injury, adult respiratory distress syndrome, disseminated intravascular coagulation, and multiple organ failure, frequently progressing to shock and death [9]. While STSS most commonly presents as necrotizing fasciitis of the extremities [10], it can occur in virtually any anatomical site or organ, including the abdominal cavity, as demonstrated by reports of peritonitis [11]. In the field of urology, cases have been documented in which epididymitis [12] or balanoposthitis [13] progressed to STSS, as well as instances where vasectomy served as the precipitating factor [14].

Regarding the diagnosis of STSS, there are no pathognomonic findings other than culture-based confirmation; therefore, it is critically important to include this condition in the differential diagnosis based on clinical presentation [15]. Standard culture methods, such as blood or urine cultures, are not invariably positive. In the present case, both blood and urine cultures were negative, and the causative organism was identified only in the retroperitoneal exudate obtained intraoperatively. The laboratory risk indicator for necrotizing fasciitis (LRINEC) score is frequently used as an adjunct in the diagnosis of necrotizing fasciitis [16]. However, the LRINEC score does not necessarily exhibit high sensitivity (67-77%) in cases of STSS, and a low score does not exclude the diagnosis [17]. In this case, the total LRINEC score was 8 points, exceeding the commonly accepted cutoff value of 6 points. As demonstrated here, STSS can also occur in the field of urology. Thus, it is essential to maintain a basic understanding of this condition and to consider it as part of the early differential diagnosis on the basis of clinical symptoms.

The most critical component of STSS management is prompt surgical intervention when indicated, particularly in cases of necrotizing fasciitis [5]. In the present case, a previously healthy 43-year-old woman developed STSS and required intensive care within three days of symptom onset due to the rapid progression of the infection. Had the lavage and drainage been delayed by even several hours or a single day, survival might have been jeopardized. When STSS is suspected, it is imperative to proceed with surgical intervention as early as possible, even if this means advancing the procedure by only a few hours.

With respect to antimicrobial therapy for STSS, penicillin G in combination with clindamycin is commonly employed. There are also reports suggesting that intravenous immunoglobulin therapy can significantly reduce mortality [5]. In this case, antimicrobial susceptibility testing of the causative organism, *S. pyogenes*, revealed high sensitivity to both agents, which allowed de-escalation from combined penicillin-clindamycin therapy to clindamycin monotherapy. Furthermore, prior reports have indicated that the use of nonsteroidal anti-inflammatory drugs in STSS may mask early symptoms and increase mortality [2]. Given that even a delay of several hours in initiating appropriate treatment can be fatal in STSS, indiscriminate use of antipyretic analgesics before diagnosis should be avoided.

## Conclusions

We present a case of STSS secondary to retroperitoneal necrotizing fasciitis, successfully managed with surgical lavage and drainage in combination with antimicrobial therapy and intensive care. STSS can manifest with a wide spectrum of clinical presentations and, although uncommon, may also occur as a retroperitoneal infection. Early consideration of STSS in the differential diagnosis is essential, particularly during the initial stages of symptom onset. Although only a few similar cases have been reported, early surgical intervention, as demonstrated in our case, appears to be a decisive factor for survival. When STSS is suspected, prompt surgical intervention should be undertaken without delay, even if this means advancing the procedure by only a few hours.

## References

[1] Stevens DL, Tanner MH, Winship J, Swarts R, Ries KM, Schlievert PM, Kaplan E (1989). Severe group A streptococcal infections associated with a toxic shock-like syndrome and scarlet fever toxin A. N Engl J Med.

[2] Bartoszko JJ, Elias Z, Rudziak P, Lo CK, Thabane L, Mertz D, Loeb M (2022). Prognostic factors for streptococcal toxic shock syndrome: systematic review and meta-analysis. BMJ Open.

[3] Fukushima S, Saito T, Iwamoto Y (2025). Trends in the growing impact of group A Streptococcus infection on public health after COVID-19 pandemic: a multicentral observational study in Okayama, Japan. Eur J Clin Microbiol Infect Dis.

[4] Inada M, Iwamoto N, Nomoto H (2024). Characteristics of streptococcal toxic shock syndrome caused by different beta-hemolytic streptococci species: a single-center retrospective study. Open Forum Infect Dis.

[5] Atchade E, De Tymowski C, Grall N, Tanaka S, Montravers P (2024). Antibiotics (Basel).

[6] Ato M, Ikebe T, Kawabata H, Takemori T, Watanabe H (2008). Incompetence of neutrophils to invasive group A streptococcus is attributed to induction of plural virulence factors by dysfunction of a regulator. PLoS ONE.

[7] Fox KL, Born MW, Cohen MA (2002). Fulminant infection and toxic shock syndrome caused by Streptococcus pyogenes. J Emerg Med.

[8] Fujimoto S, Eriguchi Y, Nakamura R (2024). Streptococcal toxic shock syndrome due to Streptococcus dysgalactiae subsp. equisimilis from retroperitoneal panniculitis during the treatment with anti-IL-6 receptor antibody: a case report. Mod Rheumatol Case Rep.

[9] Wood TF, Potter MA, Jonasson O (1993). Streptococcal toxic shock-like syndrome. The importance of surgical intervention. Ann Surg.

[10] Merola R, Negri C, Merola A (2024). Necrotizing fasciitis and streptococcal toxic shock syndrome: a case report. Cureus.

[11] Gizzatullin T (2024). Primary bacterial peritonitis in a young man: a rare manifestation of invasive group A streptococcal infection. Cureus.

[12] Walker BR, Pribble CG, Cartwright PC (2000). Invasive group A streptococcus infection of the scrotum and streptococcal toxic shock syndrome. Urology.

[13] Miyagawa T, Kawai K, Onozawa M, Hattori K, Shimazui T, Akaza H (2005). Unusual presentation of necrotizing fasciitis in a patient who had achieved long-term remission after irradiation for testicular cancer. Int J Urol.

[14] Viddeleer AC, Lycklama à Nijeholt GA (1992). Lethal Fournier's gangrene following vasectomy. J Urol.

[15] Gottlieb M, Long B, Koyfman A (2018). The evaluation and management of toxic shock syndrome in the emergency department: a review of the literature. J Emerg Med.

[16] Wong CH, Khin LW, Heng KS, Tan KC, Low CO (2004). The LRINEC (Laboratory Risk Indicator for Necrotizing Fasciitis) score: a tool for distinguishing necrotizing fasciitis from other soft tissue infections. Crit Care Med.

[17] Saijo Y, Ono S, Akiyama G (2025). The discrepancy between white blood cell and C-reactive protein levels in group a streptococcal necrotizing soft-tissue infections. Plast Reconstr Surg Glob Open.

